# Mutation landscape and intra-tumor heterogeneity of two MANECs of the esophagus revealed by multi-region sequencing

**DOI:** 10.18632/oncotarget.18678

**Published:** 2017-06-27

**Authors:** Wenqing Yuan, Zhen Liu, Wanjun Lei, Li Sun, Haijun Yang, Yu Wang, Shweta Ramdas, Xiao Dong, Ruiping Xu, Hong Cai, Jun Z. Li, Yang Ke

**Affiliations:** ^1^ Key Laboratory of Carcinogenesis and Translational Research (Ministry of Education/Beijing), Laboratory of Genetics, Peking University Cancer Hospital and Institute, Beijing 100142, PR China; ^2^ Novogene Co., LTD, Beijing 100142, PR China; ^3^ Department of Pathology, Beijing Cancer Hospital, Beijing 100142, PR China; ^4^ Anyang Cancer Hospital, Anyang 455000, PR China; ^5^ Department of Human Genetics, University of Michigan, Ann Arbor, MI 48109, USA

**Keywords:** mixed adenoneuroendocrine carcinoma (MANEC), esophageal cancer, whole-exome sequencing, intra-tumor heterogeneity, subclone evolution

## Abstract

Mixed adenoneuroendocrine carcinoma (MANEC) in the esophagus is an infrequent but highly malignant cancer with few known genomic alterations. We conducted whole-exome sequencing and whole-genome SNP genotyping for 4-6 tumor subregions and 5-6 adjacent normal tissue sites and 1-3 lymph node metastases in two esophageal MANECs to detect somatic mutations and copy number alterations, and to explore their spatial heterogeneity and underlying clonal structure. *TP53* mutation, *RB1* deletion or LOH, and *PIK3CA, PTEN, KRAS, SOX2, DVL3, TP63* amplification appeared in all regions in both tumors. Mutations falling in known cancer genes tended to show higher variant allele frequencies than those not falling in these genes in at least one of the cases. Phylogenetic analyses of the samples and underlying subclones suggested extensive migration across different tumor regions and from some regions to the lymph nodes. Lymph node metastases appeared to have been seeded by both early founder cells as well as subsequent, locally emerging daughter clones. A phenotypically normal tissue site carried most of the mutations found in neighboring tumor samples, implying field cancerization. Understanding such complex genetic heterogeneity within each patient will be important for guiding clinical decisions.

## INTRODUCTION

Primary small cell esophageal carcinoma (SCEC) was first reported by McKeown in 1952 [1] based on the presence of small, round or oval tumor cells with hyperchromatic nuclei and scanty cytoplasm. These cells are derived from the amine precursor uptake and decarboxylation (APUD) cells, and are immunohistochemically positive for neuroendocrine markers such as chromogranin A (CgA), synaptophysin (SYN), CD56 and neuron-specific enolase (NSE) [2–4]. SCEC cells mingled with differentiated squamous or glandular cells have been reported in some of the cases, suggesting that the ancestral cancerous cell might be derived from pluripotent stem cells [3–9]. In 1987, Lewin classified such mixed tumor with two components into three subtypes based on the extent of mixture: collision tumors (side-by-side pattern), combined tumors (intermingled pattern), and amphicrine tumors [10], in which the same cancer cell shows features of both types of neoplasm [2, 11]. According to the 2010 World Health Organization (WHO) classification [2, 12], small cell carcinoma mixed with a glandular or squamous component, each of which is present in at least 30% of the tumor cells, is defined as mixed adenoneuroendocrine carcinoma (MANEC). The definition of MANEC avoids the confusion caused by different names of neuroendocrine carcinoma mixed with another adenocarcinoma or squamous component [11].

MANEC in the esophagus is an infrequent disease, as most of the esophageal cancer cases belong to the two main types: squamous cell carcinoma and adenocarcinoma [13]. Although extremely rare, MANECs are highly aggressive, with poor survival outcomes [2–4, 14, 15]. Treatment of MANECs includes chemotherapy, radiation and surgical resection. However, there is no standard treatment protocol. Some MANECs benefit from the same regimens used for treating small cell lung cancer (SCLC) [16, 17], likely due to their clinical and histopathological similarity. Inactivation of *TP53* and *RB1* [18], *SOX2* amplification [19], and recurrent mutations in histone modifying genes [20] are frequent in SCLC. But little is known about genomic alterations in esophageal MANECs. To our knowledge, somatic DNA aberrations of MANECs have not been described systematically, nor has the evolutionary path of key driver mutations. Such information could lead to a deeper understanding of the biological drivers of MANECs and could potentially provide better guidance to choosing the right treatment [21].

In this study we conducted whole-exome sequencing (WES) and SNP genotyping on two esophageal MANECs and for each, we compared multiple regional samples that included apparently normal mucosae (“N”), primary tumor (“T”), and regional lymph node metastases (“LN”). We systematically analyzed the somatic variants and copy number alterations, and performed phylogenetic analysis to infer the clonal structure and evolution paths connecting the precancerous tissues to the tumor and metastases.

## RESULTS

### Clinical and histopathological features

**Figure 1:**
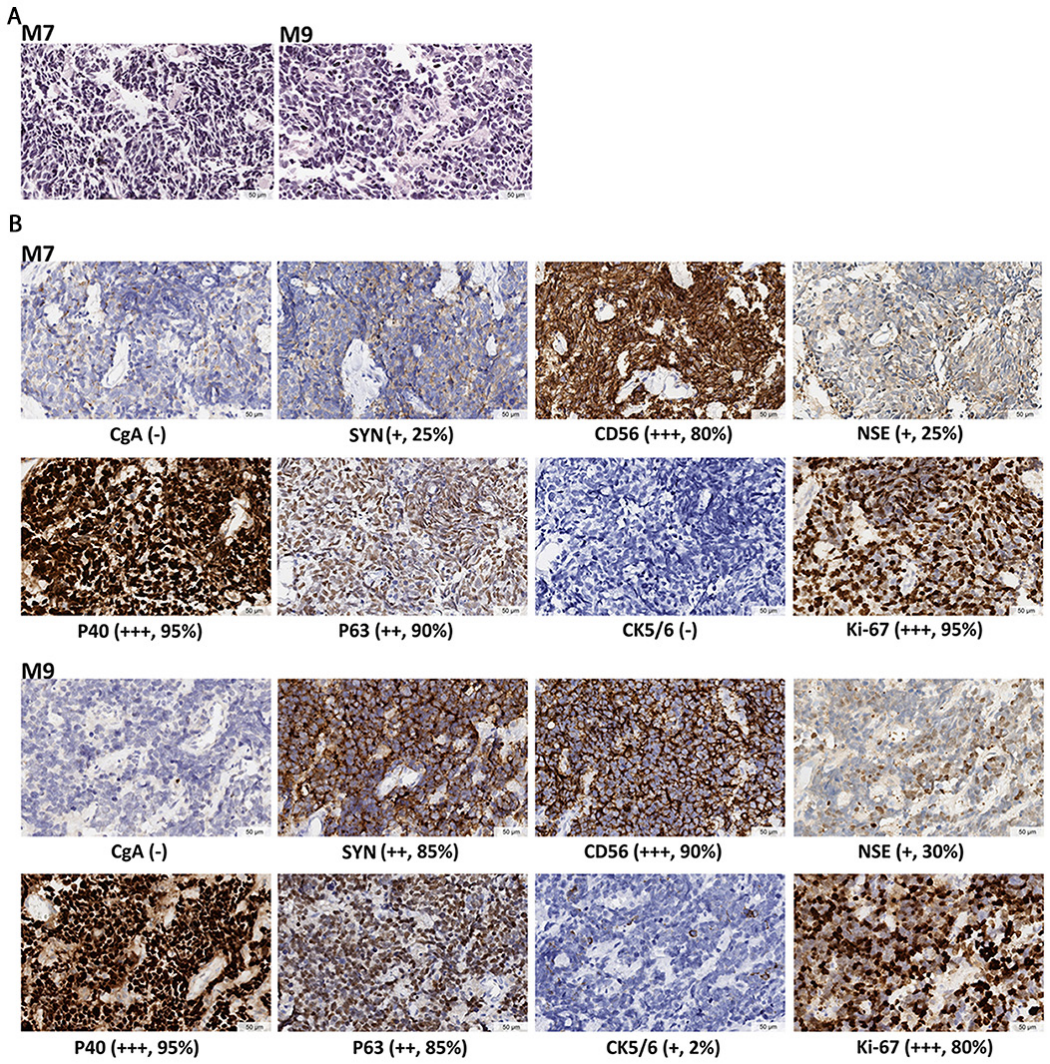
Histopathological diagnosis for the two MANECs **(A)** HE staining, showing cell features of small size, round or oval shape, hyperchromatic nuclei, and scanty cytoplasm. Nucleoli is inapparent and mitoses is prominent. **(B)** IHC staining. Neuroendocrine markers include CgA, SYN, CD56 and NSE. Squamous differentiation markers are P40, P63 and CK5/6. The marker for active proliferation is Ki-67. IHC staining demonstrates the coexistence of poorly differentiated neuroendocrine and squamous features in the same tumor. The expression levels are determined by the intensity of IHC staining. “-” indicates no stain. “+” indicates light yellow granulum and broken cell membrane. “++” indicates yellow granulum and intact cell membrane. “+++” indicates brown granulum, intact cell membrane and uniform staining. Mean positive staining percentages were calculated by averaging over ten randomly selected views for each sample.

Two patients were diagnosed with MANEC, small cell carcinoma mixed with a squamous component (amphicrine) in the Anyang Cancer Hospital (Table 1). Patient M7 was a 64-year-old female who had a 30 cm^3^ poorly differentiated tumor invading deep muscularis propria, with one para-esophageal lymph node metastasis at the time of diagnosis. Patient M9 was a 62-year-old male who had a 48 cm^3^ poorly differentiated tumor, also invading muscularis propria, with three regional lymph node metastases. Small cell morphology was observed on HE stains for both tumors (Figure 1A). IHC staining in both tumors demonstrated strong positivity for at least one of the neuroendocrine markers (CgA, SYN, CD56, NSE) in more than 80% of tumor cells and at least one of the squamous differentiation markers (P40, P63, CK5/6) in more than 95% of tumor cells (Figure 1B). The two tumors were thus classified as MANEC according to the 2010 WHO classification [12], and were graded G3 based on the positive Ki-67 index being greater than 80% (Figure 1B). Further, the diagnosis of MANEC (amphicrine) was reached for each regional tumor sample by multiple pathologists based on morphology and immunophenotype (Supplementary Figure 1). Similar analyses of the HE and IHC stain patterns confirmed that all regional “N” samples from M7 (N1-N6) and M9 (N1-N5) were diagnosed normal (Supplementary Figure 2).

**Table 1:** Clinical and histopathological characteristics

ID	Age	Sex	Tumor size (cm^3^)^a^	Location^b^	TNM^c^	Post-op follow-up	Smoke	Alcohol
**M7**	64	Female	4X3X2.5	Middle	T_2_N_1_M_0_	Died 8.6 months	No	No
**M9**	62	Male	6X4X2	Middle	T_2_N_2_M_0_	Died 19.5 months	40/day*10 yrs quit for 5 yrs	No

^a^ Tumor size was measured by length (cm) X width (cm) X height (cm).

^b^ Location of the primary tumor site is defined by the position of the upper (proximal) edge of the tumor in the esophagus.

^c^ According to AJCC: Esophageal and esophagogastric junction. In: Edge SB, Byrd DR, Compton CC, et al., eds.: AJCC Cancer Staging Manual. 7th ed. New York, NY: Springer, 2010, pp 103-15.

The samples used in this study were from surgically removed tissues, with no treatment before surgery. Platinum plus etoposide (VP-16) chemotherapy was administered to both patients after surgery. For M7, metastases developed ∼4 months after surgery in the right supraclavicular lymph node and the liver. Radiation therapy was performed, but had limited control of the metastases. The patient went into hospice care and died at 8.6 months after the initial surgery. For M9, pleural effusion developed ∼ 2 months after surgery, and suspicious bloody effusion was drained 1 month later. Cisplatin plus 5-fluorouracil pleural perfusion was performed once. The patient refused any more treatment and went home. He died at 19.5 months after the initial surgery.

### Multi-region genomic analyses of the two MANECs

**Figure 2:**
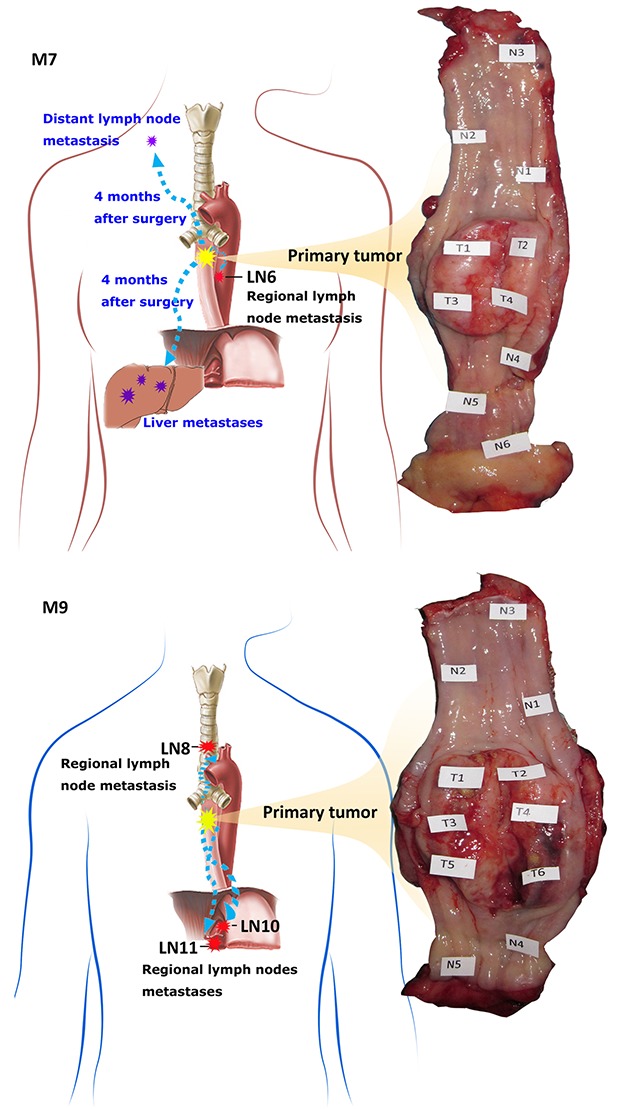
Anatomic positions of the multiple regional samples used in this study Shown is the photo of the esophagus resected by surgery, with the sampling locations marked. “N” and “T” denote spatially separated samples in adjacent apparently normal mucosae and the primary tumor. “LN” denotes lymph node metastasis. M7 has 6 “N”, 4 “T” and 1 “LN” samples collected. M9 has 5 “N”, 6 “T”, 3 “LN” samples collected.

**Figure 3:**
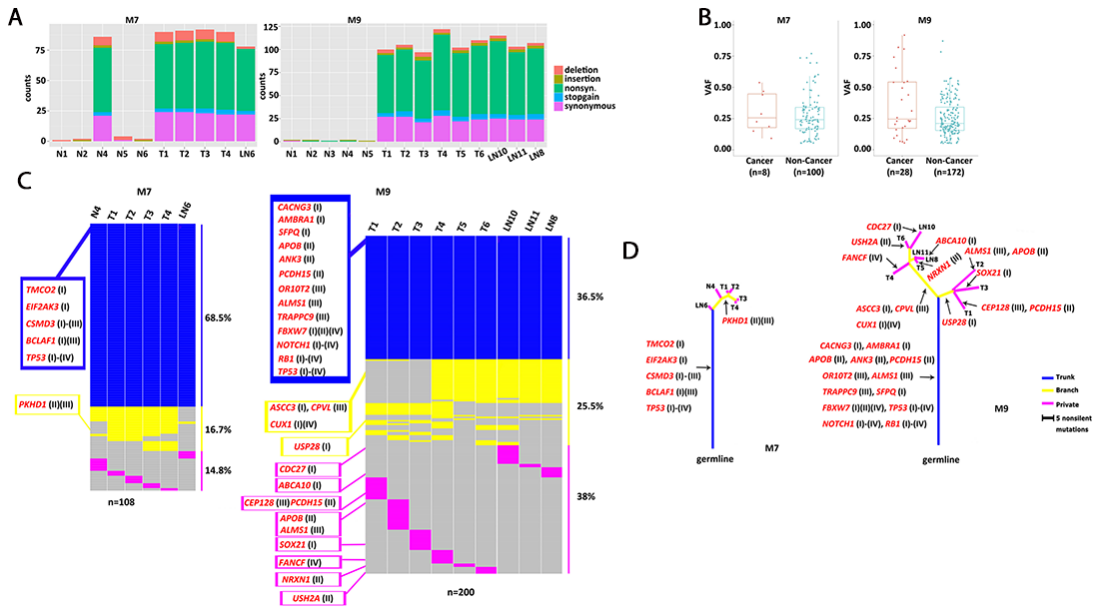
Patterns of somatic mutations and phylogenetic relationship of the regional samples **(A)** Number of five types of exonic mutations across 10 regional samples in M7 and 14 regional samples in M9. Most N samples had few mutations, except N4 in M7. **(B)** Comparison of variant allele frequency distributions for mutations falling into any of the four curated lists of cancer-related genes, versus those not falling into any of the four lists, each dot is for one mutated site. For a site that was mutated in multiple regional samples, their observed frequencies were averaged. Sometimes, a gene had more than one mutated site. The cancer vs non-cancer comparison is significant in M9 (p=0.046, Wilcoxon Test), but not significant in M7 (p=0.40, Wilcoxon Test). **(C)** Presence and absence of mutations across regional samples in each patient. Mutations were sorted from top to bottom as the trunk, branch and private mutations, which were indicated by color in blue, yellow and pink. Their proportions were indicated on the right. Cancer-related genes with nonsilent mutations were listed on the left and marked for lists I through IV. **(D)** Phylogenetic tree of the samples constructed with nonsilent mutations. The branch lengths were in proportion to the number of nonsilent mutations, including SNVs and Indels. Arrows indicate where the mutations occurred for the cancer-related genes.

To investigate the somatic mutation patterns and the extent of intra-tumor heterogeneity, we analyzed multiple regional samples of each tumor by WES and SNP genotyping. We isolated DNA from 11 and 14 regional samples from M7 and M9, respectively (Figure 2, Supplementary Table 1), including ten spatially separated regions of the tumor (M7: T1-T4; M9: T1-T6), 11 adjacent apparently normal mucosae (M7: N1-N6; M9: N1-N5), and four regional metastatic lymph nodes (M7: LN6; M9: LN8, LN10, LN11). We carried out WES with an average coverage depth of 350X (range: 264 - 396X, Supplementary Table 2A). Somatic variants were called using DNA from the blood as the control for M9, or DNA from the most distant normal tissue sample, “N3”, for M7 (see Methods for further discussionof using N3 as normal). For M7, we identified a total of 109 exonic somatic variant sites in 103 genes mutated in at least one of the ten regional samples (all except N3), including 97 single-nucleotide variants (SNVs) and 12 Indels. Of these, 68 were nonsilent substitutions, including 64 nonsynonymous and 4 stop-gain variants (Supplementary Table 3A, 3E). For M9, we identified a total of 202 exonic somatic variant sites in 190 genes over the 14 regional samples, including 188 SNVs and 14 Indels. Of these, 138 were nonsilent substitutions, including 129 nonsynonymous and 9 stop-gain variants (Supplementary Table 3B, 3E). The nonsynonymous /synonymous ratio was 2.21 (64/29) in M7 and 2.58 (129/50) in M9 (Figure 3A). C>T transition was the most frequent mutation subtype in both cancers (Supplementary Table 3F).

To validate the somatic mutations called from WES data, we designed a customized Agilent SureSelect panel targeting 68 nonsilent sites from M7 (Supplementary Table 4A) and 131 nonsilent sites from M9 (Supplementary Table 4B). We performed deep targeted re-sequencing for six M7 samples (N4, T1-T4, LN6) and eight M9 samples (T1-T6, LN8, LN11), and achieved mean coverage of 1,134X (range: 939 – 1,685X) (Supplementary Table 2B). The adjacent normal samples, with the exception of N4 in M7, tended to have very few somatic mutations in WES data (Figure 3A) and were not re-sequenced. We compared the observed somatic variant allele frequencies (VAF) between WES and DTS (Supplementary Table 4C, 4D). For the 68 targeted sites for M7, 341 mutations were called in the WES data of the six samples. 339 (99.4%) of which were recalled by DTS in the same samples (Supplementary Table 4E), with highly correlated VAF values (Pearson correlation coefficient r=0.975, p<0.001). For the 131 targeted sites for M9, 599 mutations were called in the WES data of the eight samples, 594 of which (99.2%) were recalled by DTS in the same samples (Supplementary Table 4F), with r=0.976, p<0.001.

To interpret the potential functional impact of the somatic variants (SNVs and Indels) we annotated them to highlight those falling in multiple lists of cancer-related genes, compiled from four types of sources (see Methods): (I) 635 general cancer genes, representing the union of 125 cancer drivers by Vogelstein [22], 179 significantly mutated genes by Lawrence et al [23], and 300 tumor suppressor genes (q<0.18) and 250 oncogenes (q<0.22) by Davoli et al [24]; (II) 76 ESCC-related genes from sequencing studies [25–28]; (III) 187 SCLC-related genes from sequencing studies [18–20]; and (IV) 267 genes from the *COSMIC Cancer Gene Census* and containing at least one mutation that is a missense, nonsense, frameshift or splicing site. Among the genes showing nonsilent mutations (nonsynonymous, stop-gain, stop-loss or Indels) in M7, *TP53* is in all four lists. *CSMD3* and *BCLAF1* are among the general cancer genes and SCLC-related genes. *CSMD3* is also frequently mutated in ESCC. Also notable are *TMCO2*, *EIF2AK3*, and *PKHD1* (Supplementary Table 3C). For the genes discovered in M9, *TP53*, *NOTCH1* and *RB1* are in all four lists. Other notable genes are *FBXW7*, *CUX1*, and *FANCF*. Further, *AMBRA1, CACNG3, SFPQ, USP28, ASCC3, CDC27, ABCA10* and *SOX21* are among the general cancer genes from Davoli et al [24] (Supplementary Table 3D). *PKHD1* appeared in ESCC and SCLC lists but in none of the common cancer gene lists, suggesting some molecular commonalities between ESCC and SCLC. Interestingly, this gene is mutated in M7. Further, five genes, *ALMS1, TRAPPC9, CEP128, OR10T2,* and *CPVL*, are among the SCLC genes but not in any of the common cancer gene lists, yet these genes were mutated in M9 (Supplementary Table 3D). Genes mutated in M7, M9, or either had a higher overlap rate (2.1 ∼ 5.6 fold) with ESCC- and SCLC-related genes than with the general cancer genes (Supplementary Table 3G), suggesting shared mutational profiles among MANEC, ESCC, and SCLC.

The observed variant allele frequencies vary among the somatic mutations. Those with a higher frequency likely reflect the fact that a higher proportion of the cells in the sample carried this mutation. Thus the allele frequencies can be interpreted as the relative prevalence of the subclones; and the dominant subclones are more likely to contain early-acting driver mutations than a minor subclone. Consistent with this expectation, we found that, in M9, mutations in the cancer-related genes tended to have higher VAF compared with those not falling in these genes (Figure 3B). The trend is not obvious in M7.

We detected copy number alterations (CNAs) using SNP genotyping data (see Methods). For M7, six samples (N4, T1-T4, and LN6) contained an average of 212 CNA segments (range: 148-307). Of the 1,272 segments in the six samples, 1,037 were amplifications, 88 were deletions, and 147 were copy-neutral LOH, with the median length of 2.5 Mb. Across the six samples, an average of 20% of the genome was normal diploid, and 46% was triploid. For M9, the tumor and lymph node samples (T1-T6 and LN8, LN10, LN11) contained an average of 360 CNA segments (range: 159-808). Of the 3,238 segments in the nine samples, 2,830 were amplifications, 193 were deletions, and 215 were copy-neutral LOH, with a median length of 1.7 Mb. In M9, an average of 49% of the genome was triploid, 27.3% was tetraploid, and 10% was copy-neutral LOH, indicating wide-spread impact of copy number alterations in M9 (Supplementary Table 5).

Of the 76 genes in the ESCC list, 13 also appeared in all the general cancer gene list (635 genes) and the COSMIC list (267 genes). Of these, ten reside in CNA regions in at least one tumor sample of M7, twelve in at least one tumor sample of M9. The amplification of *PIK3CA, PTEN, KRAS, SOX2, DVL3,* and *TP63* were present in all tumor samples in M7 and M9, including N4 in M7. *RB1* deletion (M7) or LOH (M9) were detected in all tumor regions. These genes were indicated in Supplementary Figure 3.

### Spatial heterogeneity and phylogenetic relationship of the samples

The analyses of spatially separated tumor samples as well as lymph node samples allowed us to assess the spatial heterogeneity of somatic alterations. Approximately 31.5% (34/108) of mutations in M7 and 63.5% (127/200) of mutations in M9 are not uniformly observed across all tumor or LN samples (Figure 3C). The presence and absence pattern of these mutations led to the construction of a “sample tree” (see Methods, Figure 3D), depicting the most likely phylogenetic relationships among the samples. In general, all the tumor samples in a patient tended to cluster together. In M7, N4 was closer to T1-4 than LN6, suggesting that it was genetically tumor-like and distinguished from T1-T4 by only a few mutations. In M9, the three LN samples clustered with T4-6, which clustered away from T1-3, indicating that the lymph nodes were preferentially seeded by cells from the lower right portion of the tumor (shown in Figure 2). Samples from the upper left side, T1-T3, did not carry mutations in *ASCC3*, *CPVL*, or *CUX1*.

We classified mutations into three classes: trunk, branch, and private. Trunk mutations were those present in all the tumor/LN samples (N4, T1-T4, LN6 in M7, and T1-T6, LN8, LN10, LN11 in M9); branch mutations were those present in some of the tumor/LN samples; while private mutations were present in only one of the samples. The relative distribution of trunk, branch, and private mutations was different between the two tumors, with M7 having a higher proportion of trunk mutations than M9. In other words, M7 had fewer additional somatic mutations beyond those carried by its most recent common founder clone, whereas M9 had a relatively greater genetic divergence among its different regions. In both samples, all three classes of mutations implicated cancer-related genes as described above and marked in Figs 3C-3D. Such results highlight that some cancer-related genes could be arriving late during tumorigenesis and acting only regionally in the tumor.

### Clonal structure of the samples

**Figure 4:**
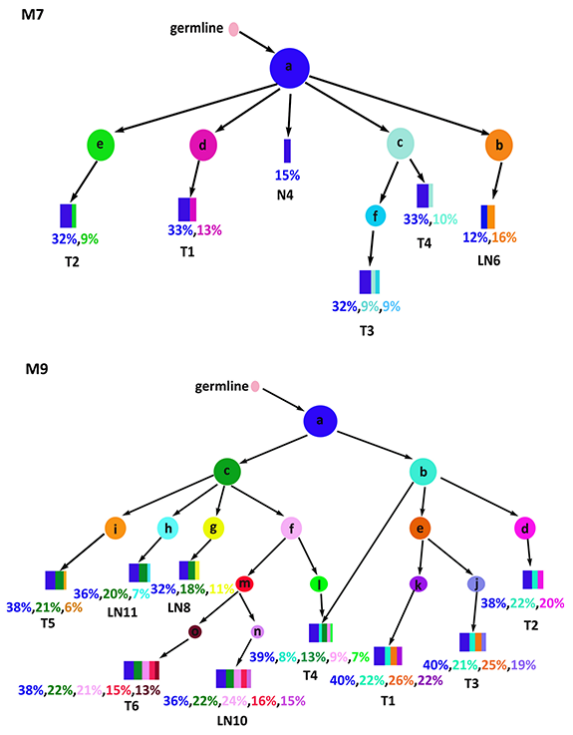
Subclones and their lineage Shown are the inferred cancer subclones, their phylogenetic relationships, and their contribution to each regional sample. Each colored circle represents a cancer clone. Relative abundance of the clones within a sample is shown by the widths of the rectangular strips drawn from the color-matched circles.

Each regional sample we analyzed still contained 10s to 100s of millions of cells and could themselves be heterogeneous, i.e., containing multiple subclones. The sample trees constructed above do not reveal the clonal composition within each sample. To gain a more accurate view of the genetic heterogeneity both within and between regional samples we investigated subclone structure and phylogenetic relationship using *LICHeE* [29]. This approach infers the clonal composition in each sample as well as the evolutionary lineage of the clones. The sample-sample relationships are replaced by the clone-clone relationships (Figure 4). In M7, a founder clone “a” is shared by N4, T1-T4, and LN6. While this clone accounted for all the cells in N4, it generated four daughter clones (b-e) by the acquisition of additional somatic variants, and these clones were observed in LN6, T4, T1, and T2, respectively. Clone-c generated its own descendant clone (clone-f) and T3 contains three subclonal populations: a, c and f. M9 showed a more complex structure: the founder clone-a produced clone-b and clone-c. Clone-b accounted for a sub-population in T4, and it further evolved into clone-d and clone-e, which eventually populated T1-T3. Clone-c led to clones f-i which, after a few levels of further diversification, accounted for T4-6 and the three LN samples, each of which consists of multiple subclones.

Such clone trees provided important clues to the source populations of lymph node metastases. In M7, LN6 contained the founder clone found in T1-4 and N4, as well as its own unique subclone (clone-b), suggesting early seeding of LN6 before the genetic diversification in T1-T4, where subsequent subclones d and e stayed in T1 and T2, respectively, and apparently did not migrate to the lymph node, hence the absence of late seeding. In M9, the three lymph nodes were seeded from both early clones (a and c) and some relatively late clones (h, g, or f-m-n), indicating extensive migration from T4-6 as well as multiple episodes of seeding. Nonetheless, such extensive migrations were not intense enough among T4-6 to conceal their heterogeneity. The T4-6 branch diverged from the T1-3 branch in the early stages, as can be seen in the early split of the clone-b and clone-c. While not marked in Figure 4, each pair of parent-child clone can be annotated by the mutation(s) distinguishing them, informing the likely driver event for the emergence of a specific child clone. In all, the clonal history of the two MANECs revealed complex sequences of mutagenesis and migration, with continuous and often early seeding of tumor cells into the adjacent lymph nodes.

## DISCUSSION

Recent years have seen a rise in the incidence of neuroendocrine carcinoma in the gastroenteropancreatic system, but much remained to be learned about its etiology [30]. This study is motivated by the opportunity to systematically document the genomic alterations in two cases of esophageal MANEC. Both patients were diagnosed MANEC with a poorly differentiated squamous component (amphicrine). Such a histopathological type is very rare, making it possible for us to report the first glimpse of the exome-wide abnormalities of this tumor type.

The two MANECs revealed 109 and 202 somatic variants in or near coding exons. As the Agilent WES platform has ∼32.3 Mb targeted coding regions, the discovered mutations correspond to an average density of 3.4-6.3 mutations/Mb, in the mid-range among other cancer types [31]. We interpreted the potential biological role of the nonsilent mutations by mapping them to four lists of cancer related genes. Lists I and IV are general driver genes summarized over many cancer types, whereas lists II and III are ESCC- and SCLC-related genes reported in recent genomic studies. The higher overlap rate of genes mutated in M7 or M9 with the ESCC- and SCLC-related genes, when compared with general cancer genes, indicates a similarity of mutational landscape shared by MANEC, ESCC, and SCLC. Genomic alterations shared by both patients including *TP53* mutations, deletion (M7) or LOH (M9) of *RB1*, amplification of *PIK3CA, PTEN, KRAS, SOX2, DVL3,* and *TP63*. Most of these have also been implicated in SCLC [18–20]. Trunk mutations in M7 and M9 highlighted five and 13 cancer-related genes, respectively, with *TP53* as the only one shared between the two (Figure 3C). One of the trunk genes in M7 is *BCLAF1*, a suppressor of BCL2. It has been reported for its general role in cancer [32–34], and was significantly mutated in SCLC [19]. A branch gene in M7 is *PKHD1*, which has one base deleted in T1-T4, but not in N4. It was frequently mutated in both ESCC [26] and SCLC [19], and is the most notable difference between N4 and its neighboring tumor samples. In M9, trunk genes *FBXW7*, *APOB, ANK3,* and *PCDH15* have been previously reported in ESCC [25, 26], consistent with M9's mixed character with a squamous cell component. *ANK3* plays a key role in cell motility and proliferation, and its down-regulation promotes cancer cell invasion [35]. Among the branch genes differentiating the T1-3 cluster and T4-6 cluster in M9, *CUX1* is a known oncogene and can make a tumor aggressive in pancreatic neuroendocrine neoplasm [36].

While most of the adjacent normal tissue samples were genetically quiescent, the exception was the normal mucosae N4 in M7. Its IHC patterns were the same as the other normal samples (Supplementary Figure 2). However, N4 had nearly the same number of somatic mutations as the T1-T4 samples (Figure 3A) and shared most of the trunk mutations (Figure 3C, 3D), and its CNA profile was similar with those of T1-T4 (Supplementary Figure 3). There was no evidence of DNA contamination in N4 and no noticeable abnormality in sample collection, DNA isolation or storage. Assuming N4's results were not due to some sample-tracking errors, our analysis suggested that N4 could be a precancerous site near the primary tumor, populated with the same ancestral founder cells that eventually developed the tumor. N4 was the closest to the primary tumor in M7 among the six apparently normal samples. Such a phenomenon of field cancerization has also been observed in non-small cell lung carcinoma [37] and colon cancer [38]. External insults such as viral infection or UV damage might cause the gradual accumulation of early genome alterations while the normal mucosa remained phenotypically normal. A recent study reported a high mutation burden, including in skin cancer-related gene, in the normal skin cells [39], serving as another example that phenotypically normal cells could already be carrying many cancer-related mutations.

Many cancer genomics studies, such as those conducted by TCGA and ICGC, focused on inter-tumor heterogeneity, and these studies have led to refined classifications of cancer subtypes. Meanwhile, it has been recognized since half a century ago that a typical cancer is not a homogeneous tissue [40]. Understanding intra-tumor heterogeneity for each patient is therefore the key to his/her successful personalized treatment. This study belongs to those that closely examine intra-tumor heterogeneity, treating each tumor as a self-contained evolutionary system, and interpreting the data in terms of clonal evolution in space and time [41–45].

We evaluated clonal heterogeneity from two related and complementary perspectives. First, we designed our study with the explicit goal to compare spatially separated sectors of each tumor, and to rank the mutations by their range of spatial distribution. The three classes of mutations thus defined, trunk, branch, and private, are then used to estimate their likely roles in early or late tumorigenesis. The phylogenetic tree of the M9 samples showed the divergence between upper-left versus lower-right samples, and identified the lower-right portion as the likely source of the three lymph node metastases. Second, while the regional samples are different from each other, each of them contained further subclone heterogeneity within itself. From this second perspective, we took advantage of our deep sequencing depth—300X for the exome and 1,000X for the targeted resequencing—to rank cancer-related genes by their observed somatic mutation frequencies. Interestingly, known cancer genes tended to carry mutations of higher clonal frequencies (Figure 3B), suggesting their action during early stages of cancer evolution.

The genomic analysis of the two MANECs in this study revealed multiple genes that were plausibly involved in the development of this rare tumor. The candidate mutations showed varying degrees of spatial range and within-region clonality. The exact driver mutation(s) acting in every stage, from the tumor cell transformation, proliferation, local invasion to adjacent normal mucosae, to long-range migration to lymph nodes, remain unclear and require future profiling of additional MANEC cases as well as targeted functional studies. Nonetheless, as a first systematic report of genomic aberrations in esophageal MANECs we leveraged deep multi-region sequencing to catalog somatic mutations and copy number alterations, annotate their spatial distribution and clonality. The data reflected complex evolutionary events taken place in the past, including a possible example of field cancerization and evidence of multiple waves of seeding from the primary tissue to the lymph nodes. As such, migration seemed extensive and ongoing, connecting multi-clonal populations at both the source site and the destination. Fully documenting this history will be necessary for predicting the future clinical course of each individual MANEC.

## MATERIALS AND METHODS

### Sample collection and histopathological diagnosis

Two patients, having undergone esophagogastrectomy and radical resection of esophageal cancer, respectively, in the Anyang Cancer Hospital (in Henan province, China) were diagnosed with MANEC. The study protocol was reviewed and approved by the Peking University IRB (IRB00001052 -15019). Informed consent was obtained from each patient before surgery. We collected each patient’s peripheral blood before surgery, and adjacent apparently normal esophageal mucosae (“N”), primary tumor tissues (“T”) as well as lymph nodes (“LN”) immediately after surgery. “N” samples were collected along the esophagus at a sequential interval of 1 cm, 2 cm from the tumor margin, and at both ends of the resected esophagus. Multi-region “T” samples were separated from each other at a distance of ≥1cm. Each “N” and “T” sample was further cut into two 5mm X 5mm X 5mm tissue blocks. One was intended for DNA extraction and was stored at -80°C immediately after cutting. The other one and the “LN” samples were formalin fixed immediately after surgery and paraffin embedded 24 hours later. Hematoxylin-eosin (HE) staining was performed on all of the “N”, “T”, “LN” samples. Immunohistochemical (IHC) staining were performed on “N” and “T” samples. CgA, SYN, CD56 and NSE were selected as neuroendocrine markers. P40, P63 and CK5/6 were used to indicate squamous differentiation. Ki-67 was used to indicate the level of proliferation. Two experienced pathologists from the Anyang Cancer Hospital and the Beijing Cancer Hospital were invited to give the pathological diagnosis separately. Tumor content was estimated by averaging over ten randomly selected views of HE and IHC patterns for each multi-region sample. All “N” samples, “T” samples with tumor content ≥60%, “LN” samples with tumor content ≥40% were selected for DNA extraction using QIAGEN DNeasy Blood & Tissue Kits or QIAamp DNA FFPE Tissue Kits while following manufacturer’s protocols. Rigorous standard operation procedure was followed to avoid cell contamination. DNA was examined by agarose gel electrophoresis for integrity and by NanoDrop (ND-1000) for purity and amount. The blood from M7 yielded too little DNA and was excluded from WES. The 11-sample set for M7 WES contained N1-N6, T1-T4, and LN6. The 15-sample set for M9 WES contained blood, N1-N5, T1-T6 and LN8, LN10, LN11.

### Multi-region whole-exome sequencing and mutation calling

WES utilized Agilent SureSelect Human All Exon V5 kit for exome capture, and was performed on the Illumina HiSeq 4000 sequencing platform using a 150 nt paired-end strategy. Barcoded samples were sequenced in parallel and reached an average sequencing depth of 350.4X (ranging from 264.3X to 395.8X) (for other sequencing quality measures see Supplementary Table 2A). Blood was used as the germline control for the 14 multi-region samples from M9. Unfortunately, we couldn’t obtain enough blood DNA from M7 and we chose the farthest, histopathological diagnosed normal sample N3 as the matched control for the other 10 multi-region samples from M7. We confirmed that N3 has a normal diploid genome based on *ASCAT* [46] analysis. Raw reads were mapped to the human genome reference provided by 1000 Genomes Project builder V37 (human_g1k_v37. fasta) using the Burrows-Wheeler Aligner (*BWA*) [47]. The aligned reads were further processed following the GATK Best Practices workflow (https://software.broadinstitute.org/gatk/best-practices/). Duplicate reads were marked and removed by picard-tools-1.105. Local realignment was performed based on the “Mills_and_1000G_gold_standard. indels.b37.sites.vcf”. Base quality was recalibrated based on the empirical base quality. Somatic single-nucleotide substitutions (SNV) and Indels were called by *MuTect* 1.1.4 [48] and *VarScan* 2.3.7 [49], respectively, using default parameters. We further filtered the *MuTect* SNV callset by keeping those with the alternative allele frequency in control samples ≤0.05, and alternative allele frequency of “T”, “N” and “LN” samples >0.05. Indels were further filtered by p-value <0.01 and alternative allele frequency of “T”, “N” and “LN” samples >0.1. We then used processSomatic in *VarScan* to obtain high confidence somatic Indels. Lastly, we removed variants with more than two alternative allele reads in control samples. ANNOVAR [50] was used for annotation of nonsynonymous, stop-gain and stop-loss variants.

Cancer-related genes were defined based on four types of sources: (I) a list of 635 cancer genes, compiled by merging those nominated by Vogelstein [22], Lawrence et al. [23], and the top 300 tumor suppressor genes (q<0.18) and top 250 oncogenes (q<0.22) in the study by Davoli et al. [24]; (II) 76 ESCC-related genes from four sequencing-based studies [25–28]; (III) 187 SCLC-related genes from three sequencing studies [18–20] and (IV) 616 genes from the *COSMIC Cancer Gene Census* (downloaded from http://cancer.sanger.ac.uk/census, 03/06/2017), then selecting the subset of 267 genes with at least one of the following four mutation types: missense, frameshift, nonsense, and splice site.

### Validation by deep targeted sequencing (DTS)

We designed an Agilent’s SureSelect customized panel to validate nonsilent somatic SNVs. All “T” and “LN” samples with enough DNA and “N” samples with at least one nonsilent mutation in cancer-related genes were selected. For patient M7, T1-T4, N4 and LN6 were included in the DTS panel with N3 as control. For patient M9, T1-T6, LN8 and LN11 were included with blood as control. We targeted the 50 bp flanking regions of each nonsilent mutation and used the 2X tiling density to cover the 101 bp region. DTS was performed on the Illumina HiSeq 4000 platform. The average coverage depth is 1,134.3 X (ranging from 939.1 X to 1,684.8 X) (Supplementary Table 2B). Variant calling process was the same as WES.

### Copy number alteration analysis

We conducted SNP genotyping on Illumina Human OmniZhongHua-8 BeadChips on the 11-sample set for M7 and the 15-sample set for M9. Allelic intensity data including B Allele Frequency (BAF) and Log R Ratio (LRR) were generated by the Illumina GenomeStudio using default parameters. *ASCAT* [46] was used to infer the allele-specific copy number at each SNP. We used blood as germline control in M9 and used the mean value of N1, N2, N3, N5 and N6 as the control in M7 because of the lack of blood DNA.

### Intra-tumor genetic heterogeneity and clonal evolution analysis

We first plotted sample trees based on the presence or absence of somatic SNVs and Indels called by WES for both M7 and M9. The branch distance was calculated based on the Manhattan distances between pairs of multi-region samples. Neighbor-Joining (NJ) trees were drawn using R packages *ape* [51] (version 3.3) and *phangorn* [52] (version 1.99-14) with each leaf representing a sample. Trunk, branch, private mutations were defined as mutations in all the samples, some of the samples, and only one sample, respectively.

To further explore the evolution of tumor cells, we drew subclone trees with each node representing a clone or subclone. Multi-sample cell lineage was reconstructed by *LICHeE* [29] using the observed allele frequencies. *LICHeE* employs a two-step clustering procedure and uses allele frequency information of all mutations across all samples to infer the number of clones. It builds the most likely phylogenetic relationships among clones as well as the proportion of each clone in each regional samples

### Data accessibility

Bam files of WES and DTS have been deposited into the Sequence Read Archive, under accession number SRP079168.

## SUPPLEMENTARY MATERIALS FIGURES AND TABLES













## References

[1] McKeown F (1952). Oat-cell carcinoma of the oesophagus. J Pathol Bacteriol.

[2] La Rosa S, Marando A, Sessa F, Capella C (2012). Mixed adenoneuroendocrine carcinomas (MANECs) of the gastrointestinal tract: an update. Cancers (Basel).

[3] Zhu Y, Qiu B, Liu H, Li Q, Xiao W, Hu Y, Liu M (2014). Primary small cell carcinoma of the esophagus: review of 64 cases from a single institution. Dis Esophagus.

[4] Yun JP, Zhang MF, Hou JH, Tian QH, Fu J, Liang XM, Wu QL, Rong TH (2007). Primary small cell carcinoma of the esophagus: clinicopathological and immunohistochemical features of 21 cases. BMC Cancer.

[5] Fukunaga Y, Hirata S, Tanimura S, Okawa K, Higashino M, Inoue T, Kobayashi Y (2000). Superficial undifferentiated small cell carcinoma of the esophagus showing an interesting growing pattern in histology. Hepatogastroenterology.

[6] Takubo K, Nakamura K, Sawabe M, Arai T, Esaki Y, Miyashita M, Mafune K, Tanaka Y, Sasajima K (1999). Primary undifferentiated small cell carcinoma of the esophagus. Hum Pathol.

[7] Ho KJ, Herrera GA, Jones JM, Alexander CB (1984). Small cell carcinoma of the esophagus: evidence for a unified histogenesis. Hum Pathol.

[8] Yang LL, Sun X, Zou YB, Meng XW (2014). Small cell type neuroendocrine carcinoma colliding with squamous cell carcinoma at esophagus. Int J Clin Exp Pathol.

[9] Nayal B, Vasudevan G, Rao AC, Kudva R, Valliathan M, Mathew M, Rao L (2015). Primary small cell carcinoma of the esophagus - an eight year retrospective study. J Clin Diagn Res.

[10] Lewin K (1987). Carcinoid tumors and the mixed (composite) glandular-endocrine cell carcinomas. Am J Surg Pathol.

[11] Li Y, Yau A, Schaeffer D, Magliocco A, Gui X, Urbanski S, Waghray R, Owen D, Gao ZH (2011). Colorectal glandular-neuroendocrine mixed tumor: pathologic spectrum and clinical implications. Am J Surg Pathol.

[12] Bosman FT, Carneiro F, Hruban RH, Theise ND

[13] Zeng H, Zheng R, Zhang S, Zuo T, Xia C, Zou X, Chen W (2016). Esophageal cancer statistics in China, 2011: estimates based on 177 cancer registries. Thorac Cancer.

[14] Maru DM, Khurana H, Rashid A, Correa AM, Anandasabapathy S, Krishnan S, Komaki R, Ajani JA, Swisher SG, Hofstetter WL (2008). Retrospective study of clinicopathologic features and prognosis of high-grade neuroendocrine carcinoma of the esophagus. Am J Surg Pathol.

[15] Brathwaite S, Rock J, Yearsley MM, Bekaii-Saab T, Wei L, Frankel WL, Hays J, Wu C, Abdel-Misih S (2016). Mixed adeno-neuroendocrine carcinoma: an aggressive clinical entity. Ann Surg Oncol.

[16] Sengoz M, Abacioglu U, Salepci T, Eren F, Yumuk F, Turhal S (2003). Extrapulmonary small cell carcinoma: multimodality treatment results. Tumori.

[17] Araki T, Takashima A, Hamaguchi T, Honma Y, Iwasa S, Okita N, Kato K, Yamada Y, Hashimoto H, Taniguchi H, Kushima R, Nakao K, Boku N (2016). Amrubicin in patients with platinum-refractory metastatic neuroendocrine carcinoma and mixed adenoneuroendocrine carcinoma of the gastrointestinal tract. Anticancer Drugs.

[18] George J, Lim JS, Jang SJ, Cun Y, Ozretic L, Kong G, Leenders F, Lu X, Fernandez-Cuesta L, Bosco G, Muller C, Dahmen I, Jahchan NS (2015). Comprehensive genomic profiles of small cell lung cancer. Nature.

[19] Rudin CM, Durinck S, Stawiski EW, Poirier JT, Modrusan Z, Shames DS, Bergbower EA, Guan Y, Shin J, Guillory J, Rivers CS, Foo CK, Bhatt D (2012). Comprehensive genomic analysis identifies SOX2 as a frequently amplified gene in small-cell lung cancer. Nat Genet.

[20] Peifer M, Fernandez-Cuesta L, Sos ML, George J, Seidel D, Kasper LH, Plenker D, Leenders F, Sun R, Zander T, Menon R, Koker M, Dahmen I (2012). Integrative genome analyses identify key somatic driver mutations of small-cell lung cancer. Nat Genet.

[21] McGranahan N, Swanton C (2015). Biological and therapeutic impact of intratumor heterogeneity in cancer evolution. Cancer Cell.

[22] Vogelstein B, Papadopoulos N, Velculescu VE, Zhou S, Diaz LA, Kinzler KW (2013). Cancer genome landscapes. Science.

[23] Lawrence MS, Stojanov P, Mermel CH, Robinson JT, Garraway LA, Golub TR, Meyerson M, Gabriel SB, Lander ES, Getz G (2014). Discovery and saturation analysis of cancer genes across 21 tumour types. Nature.

[24] Davoli T, Xu AW, Mengwasser KE, Sack LM, Yoon JC, Park PJ, Elledge SJ (2013). Cumulative haploinsufficiency and triplosensitivity drive aneuploidy patterns and shape the cancer genome. Cell.

[25] Zhang L, Zhou Y, Cheng C, Cui H, Cheng L, Kong P, Wang J, Li Y, Chen W, Song B, Wang F, Jia Z, Li L (2015). Genomic analyses reveal mutational signatures and frequently altered genes in esophageal squamous cell carcinoma. Am J Hum Genet.

[26] Gao YB, Chen ZL, Li JG, Hu XD, Shi XJ, Sun ZM, Zhang F, Zhao ZR, Li ZT, Liu ZY, Zhao YD, Sun J, Zhou CC (2014). Genetic landscape of esophageal squamous cell carcinoma. Nat Genet.

[27] Lin DC, Hao JJ, Nagata Y, Xu L, Shang L, Meng X, Sato Y, Okuno Y, Varela AM, Ding LW, Garg M, Liu LZ, Yang H (2014). Genomic and molecular characterization of esophageal squamous cell carcinoma. Nat Genet.

[28] Song Y, Li L, Ou Y, Gao Z, Li E, Li X, Zhang W, Wang J, Xu L, Zhou Y, Ma X, Liu L, Zhao Z (2014). Identification of genomic alterations in oesophageal squamous cell cancer. Nature.

[29] Popic V, Salari R, Hajirasouliha I, Kashef-Haghighi D, West RB, Batzoglou S (2015). Fast and scalable inference of multi-sample cancer lineages. Genome Biol.

[30] Ilett EE, Langer SW, Olsen IH, Federspiel B, Kjaer A, Knigge U (2015). Neuroendocrine carcinomas of the gastroenteropancreatic aystem: a comprehensive review. Diagnostics (Basel).

[31] Lawrence MS, Stojanov P, Polak P, Kryukov GV, Cibulskis K, Sivachenko A, Carter SL, Stewart C, Mermel CH, Roberts SA, Kiezun A, Hammerman PS, McKenna A (2013). Mutational heterogeneity in cancer and the search for new cancer-associated genes. Nature.

[32] Shen B, Tan M, Mu X, Qin Y, Zhang F, Liu Y, Fan Y (2016). Upregulated SMYD3 promotes bladder cancer progression by targeting BCLAF1 and activating autophagy. Tumour Biol.

[33] Shao AW, Sun H, Geng Y, Peng Q, Wang P, Chen J, Xiong T, Cao R, Tang J (2016). Bclaf1 is an important NF-kappaB signaling transducer and C/EBPbeta regulator in DNA damage-induced senescence. Cell Death Differ.

[34] Zhou X, Li X, Cheng Y, Wu W, Xie Z, Xi Q, Han J, Wu G, Fang J, Feng Y (2014). BCLAF1 and its splicing regulator SRSF10 regulate the tumorigenic potential of colon cancer cells. Nat Commun.

[35] Wang T, Abou-Ouf H, Hegazy SA, Alshalalfa M, Stoletov K, Lewis J, Donnelly B, Bismar TA (2016). Ankyrin G expression is associated with androgen receptor stability, invasiveness, and lethal outcome in prostate cancer patients. J Mol Med (Berl).

[36] Krug S, Kuhnemuth B, Griesmann H, Neesse A, Muhlberg L, Boch M, Kortenhaus J, Fendrich V, Wiese D, Sipos B, Friemel J, Gress TM, Michl P (2014). CUX1: a modulator of tumour aggressiveness in pancreatic neuroendocrine neoplasms. Endocr Relat Cancer.

[37] Jakubek Y, Lang W, Vattathil S, Garcia M, Xu L, Huang L, Yoo SY, Shen L, Lu W, Chow CW, Weber Z, Davies G, Huang J (2016). Genomic landscape established by allelic imbalance in the cancerization field of a normal appearing airway. Cancer Res.

[38] Park SK, Song CS, Yang HJ, Jung YS, Choi KY, Koo DH, Kim KE, Jeong KU, Kim HO, Kim H, Chun HK, Park DI (2016). Field cancerization in sporadic colon cancer. Gut Liver.

[39] Martincorena I, Roshan A, Gerstung M, Ellis P, Van Loo P, McLaren S, Wedge DC, Fullam A, Alexandrov LB, Tubio JM, Stebbings L, Menzies A, Widaa S (2015). Tumor evolution. High burden and pervasive positive selection of somatic mutations in normal human skin. Science.

[40] Nowell PC (1976). The clonal evolution of tumor cell populations. Science.

[41] Morrissy AS, Garzia L, Shih DJ, Zuyderduyn S, Huang X, Skowron P, Remke M, Cavalli FM, Ramaswamy V, Lindsay PE, Jelveh S, Donovan LK, Wang X (2016). Divergent clonal selection dominates medulloblastoma at recurrence. Nature.

[42] Kim H, Zheng S, Amini SS, Virk SM, Mikkelsen T, Brat DJ, Grimsby J, Sougnez C, Muller F, Hu J, Sloan AE, Cohen ML, Van Meir EG (2015). Whole-genome and multisector exome sequencing of primary and post-treatment glioblastoma reveals patterns of tumor evolution. Genome Res.

[43] Yates LR, Gerstung M, Knappskog S, Desmedt C, Gundem G, Van Loo P, Aas T, Alexandrov LB, Larsimont D, Davies H, Li Y, Ju YS, Ramakrishna M (2015). Subclonal diversification of primary breast cancer revealed by multiregion sequencing. Nat Med.

[44] Gerlinger M, Horswell S, Larkin J, Rowan AJ, Salm MP, Varela I, Fisher R, McGranahan N, Matthews N, Santos CR, Martinez P, Phillimore B, Begum S (2014). Genomic architecture and evolution of clear cell renal cell carcinomas defined by multiregion sequencing. Nat Genet.

[45] Bashashati A, Ha G, Tone A, Ding J, Prentice LM, Roth A, Rosner J, Shumansky K, Kalloger S, Senz J, Yang W, McConechy M, Melnyk N (2013). Distinct evolutionary trajectories of primary high-grade serous ovarian cancers revealed through spatial mutational profiling. J Pathol.

[46] Van Loo P, Nordgard SH, Lingjaerde OC, Russnes HG, Rye IH, Sun W, Weigman VJ, Marynen P, Zetterberg A, Naume B, Perou CM, Borresen-Dale AL, Kristensen VN (2010). Allele-specific copy number analysis of tumors. Proc Natl Acad Sci U S A.

[47] Li H, Durbin R (2009). Fast and accurate short read alignment with Burrows-Wheeler transform. Bioinformatics.

[48] Cibulskis K, Lawrence MS, Carter SL, Sivachenko A, Jaffe D, Sougnez C, Gabriel S, Meyerson M, Lander ES, Getz G (2013). Sensitive detection of somatic point mutations in impure and heterogeneous cancer samples. Nat Biotechnol.

[49] Koboldt DC, Zhang Q, Larson DE, Shen D, McLellan MD, Lin L, Miller CA, Mardis ER, Ding L, Wilson RK (2012). VarScan 2: somatic mutation and copy number alteration discovery in cancer by exome sequencing. Genome Res.

[50] Wang K, Li M, Hakonarson H (2010). ANNOVAR: functional annotation of genetic variants from high-throughput sequencing data. Nucleic Acids Res.

[51] Paradis E, Claude J, Strimmer K (2004). APE: analyses of phylogenetics and evolution in R language. Bioinformatics.

[52] Schliep KP (2011). phangorn: phylogenetic analysis in R. Bioinformatics.

