# Supplementary material for: Mutation landscape and intra-tumor heterogeneity of two MANECs of the esophagus revealed by multi-region sequencing
Source: Oncotarget. 2017 Jun 27;8(41):69610–21. doi: 10.18632/oncotarget.18678 (PMC5642503; doi:10.18632/oncotarget.18678)
Supplement: Supplementary file 1 [file oncotarget-08-69610-s001.pdf]

Mutation landscape and intra-tumor heterogeneity of two MANECs of the esophagus revealed by multi-region sequencing

SUPPLEMENTARY MATERIALS

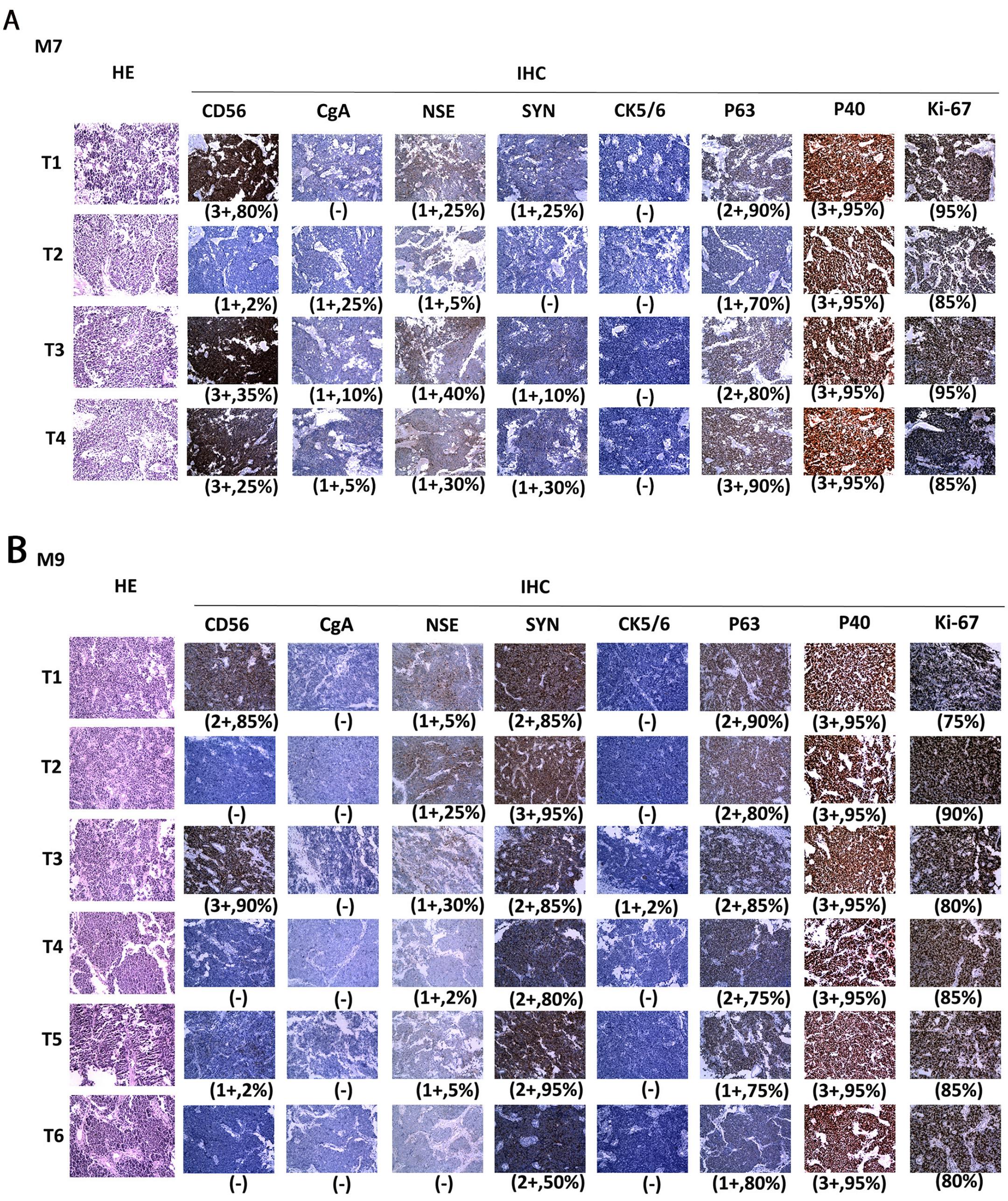

Supplementary Figure 1: HE and IHC staining of each regional tumor samples **(A)** for T1-T4 in M7; **(B)** for T1-T6 in M9.

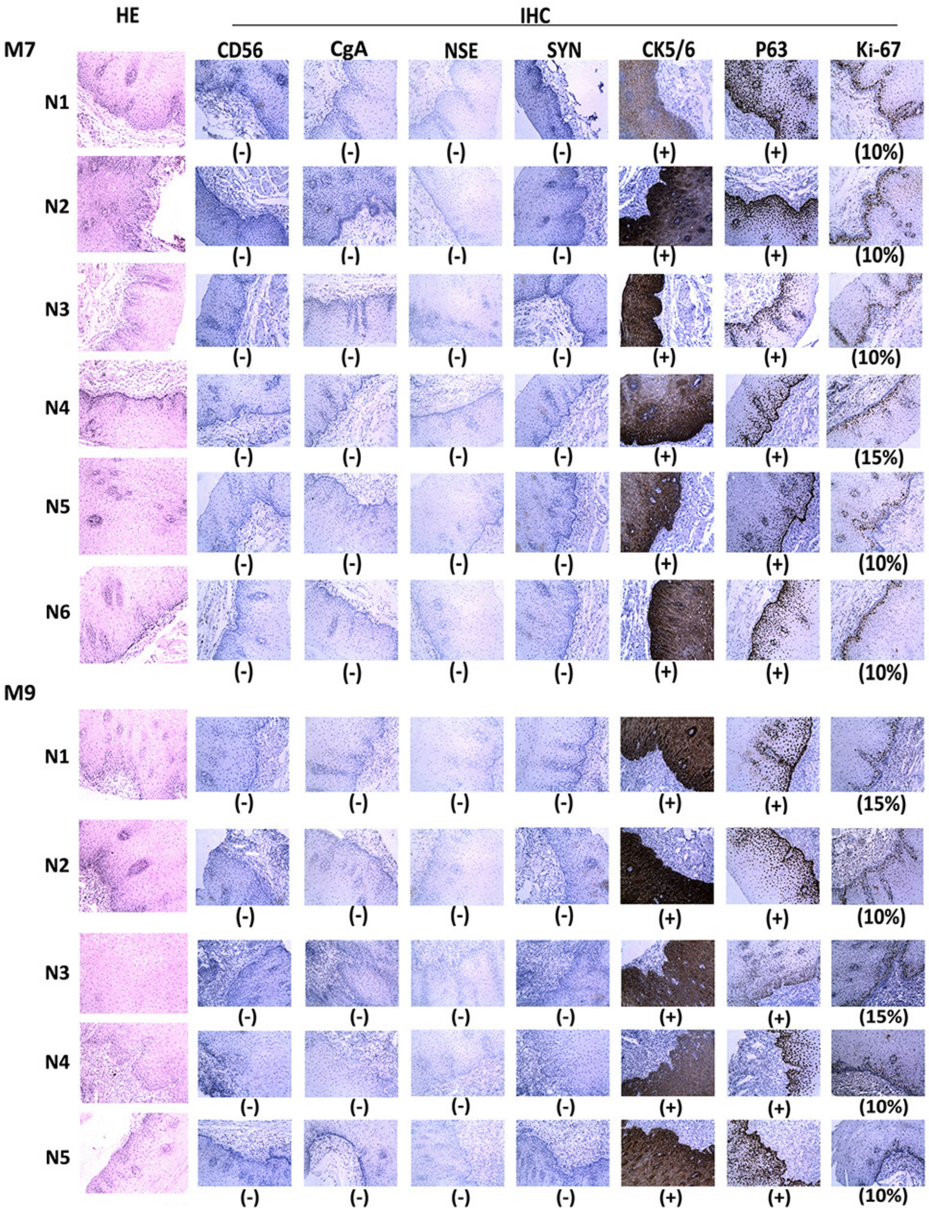

Supplementary Figure 2: HE and IHC staining of N samples. All normal samples in both cancers demonstrated a normal histochemical phenotype.

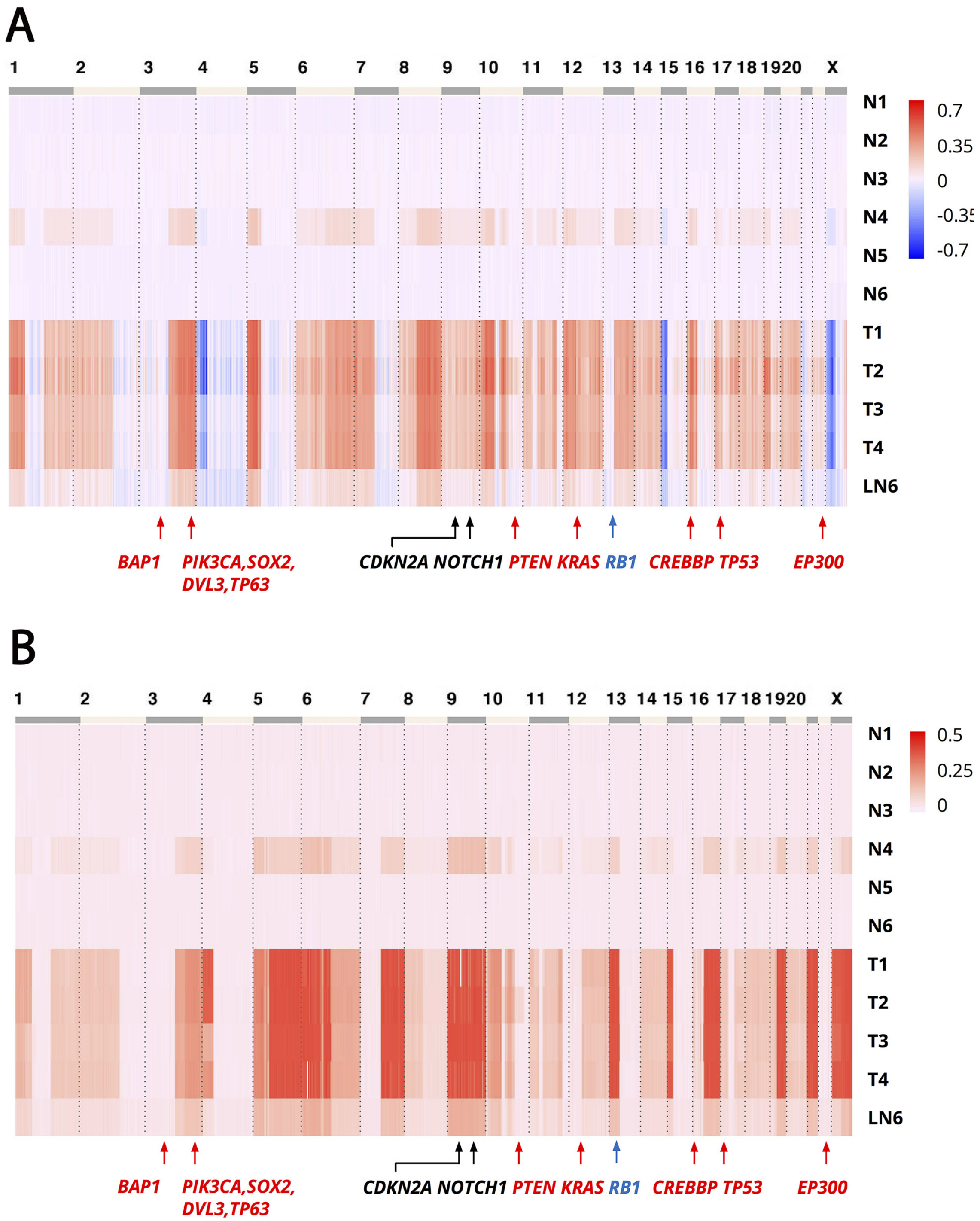

Supplementary Figure 3: Copy number alterations in M7 and M9. Shown are LRR **(A** and **C)** and BAF **(B** and **D)** patterns in 1,000-SNP bins for all regional samples in M7 and M9. Cancer-related genes or ESCC genes mentioned in the main text were indicated by arrows, with text colors showing the CNA status (red: amplification; blue: deletion; black: amplified or copy-neutral LOH). (*Continued*)

C

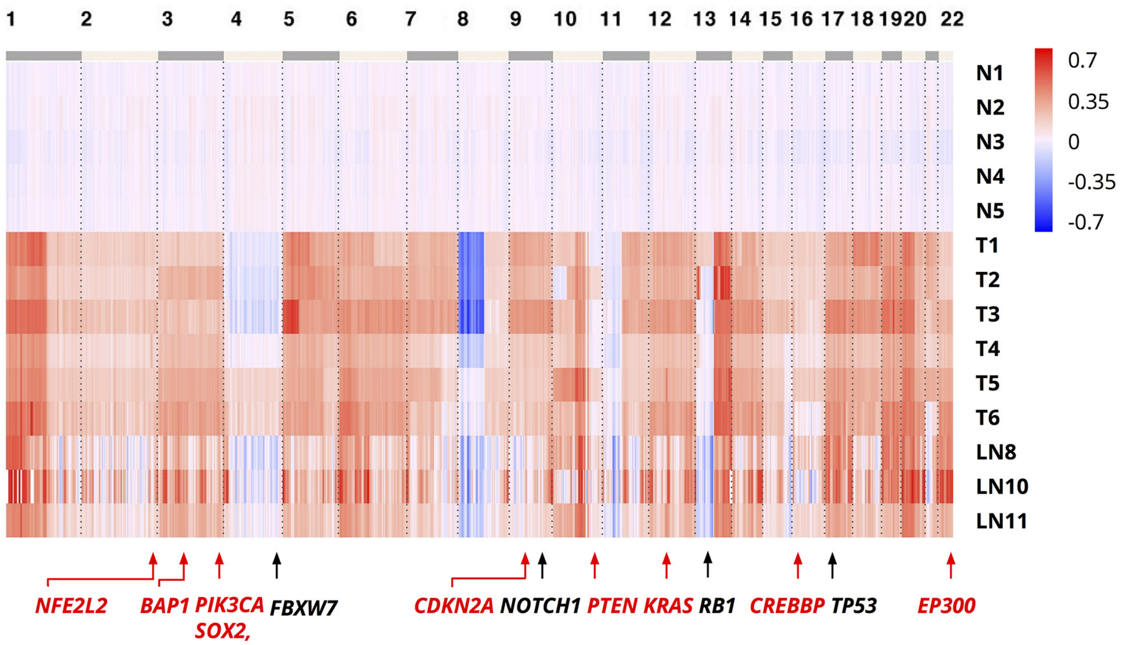

D

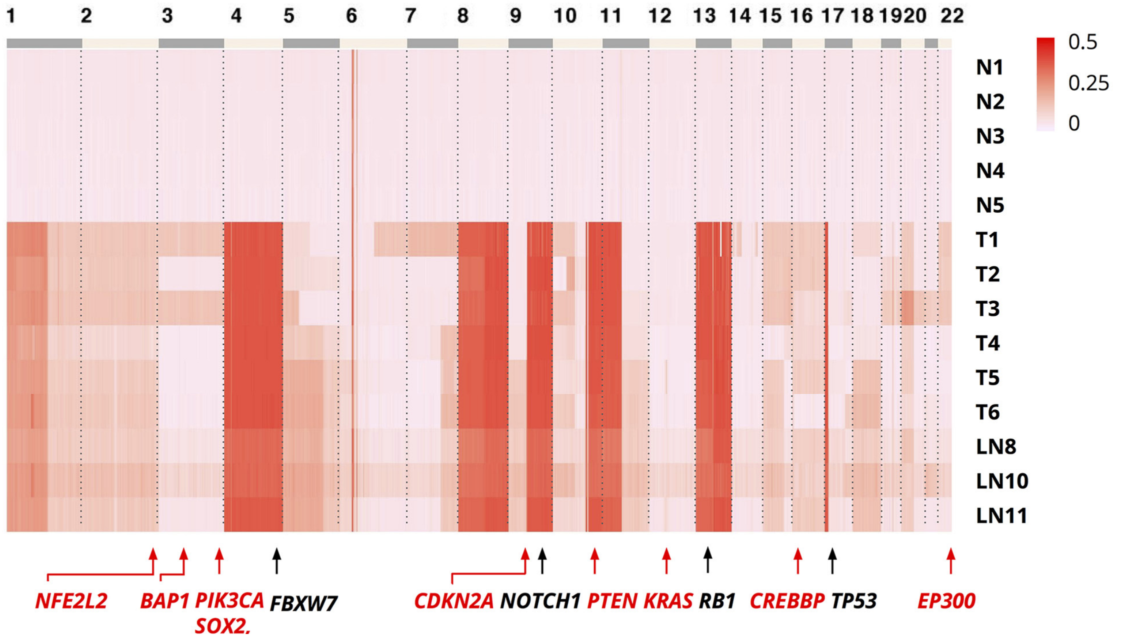

Supplementary Figure 3: ***(Continued)*** **Copy number alterations in M7 and M9.** Shown are LRR (a and c) and BAF (b and d) patterns in 1,000-SNP bins for all regional samples in M7 and M9. Cancer-related genes or ESCC genes mentioned in the main text were indicated by arrows, with text colors showing the CNA status (red: amplification; blue: deletion; black: amplified or copy-neutral LOH).

Supplementary Table 1: Characteristics of the regional samples. Shown are the concentration (ng/ul) and 260/280 ratio of the DNA samples and the pathologic diagnosis as well as percentage of the squamous and neuroendocrine components for the 11 M7 and 14 M9 samples.

See Supplementary File 1

Supplementary Table 2A: Sequencing quality metrics for whole-exome sequencing of 26 samples. Column A is the sample name. From columns B to Q are: total number of reads (B), number of duplicate reads (C), percentage of duplicate reads (D), number of total reads that mapped to the reference genome (E), percentage of mapped reads (F), number of reads that mapped to the reference genome and in the same direction (G), percentage of properly mapped reads (H), total bases mapped to exome (I), the valid data size that mapped to reference genome (Mb) (J), total reads that mapped to the reference exome (K), the average sequencing depth that mapped to the reference exome (L), the coverage length of target region (M), the percentage of covered targeted region (N), the percentage of bases with depth > 20X (O), >10X (P), >4X (Q) in targeted region.

See Supplementary File 2

Supplementary Table 2B: Sequencing quality metrics for deep targeted re-sequencing of 14 samples. Columns are the same as in Supplementary Table 2A.

See Supplementary File 2

Supplementary Table 3A: 109 exonic mutations in M7. Shown are their location, gene annotation, functional properties and overlap with multiple lists of cancer-related genes. Columns A-B are the Entrez Gene ID and Refseq gene names. Columns C-E are the chromosome, start, and end position. Columns F-H are the nucleotide change (from germline to somatic), substitution type (transition or transversion), and annotated function change, including synonymous SNV, nonsynonymous SNV, stop-gain and Indels. Columns I-R are the 10 regional samples in M7. Columns S-Y are general cancer genes defined by three studies. Columns Z-AC are ESCC related genes from four studies, and Columns AD-AF are SCLC related genes from three studies. Columns AG-AM are the COSMIC genes from the Cancer Gene Census.

See Supplementary File 3

Supplementary Table 3B: 202 exonic mutations in M9. Content organization is similar to that in S3A, except that columns I-V are the 14 regional samples in M9.

See Supplementary File 3

Supplementary Table 3C: Candidate driver mutations in M7. Shown are exonic nonsilent mutations in M7 that fall into at least one of the four lists of cancer-related genes. Format is the same as in S3A.

See Supplementary File 3

Supplementary Table 3D: Candidate driver mutations in M9. Shown are exonic nonsilent mutations in M9 that fall into at least one of the four lists of cancer-related genes. Format is the same as in S3A.

See Supplementary File 3

Supplementary Table 3E: Distribution of mutation counts. Counts of five types of exonic mutations across 10 regional samples in M7 and 14 regional samples in M9. These data were plotted in Figure 3A.

See Supplementary File 3

Supplementary Table 3F: Distribution of substitution counts. Counts of two transition and four transversion types across 10 regional samples in M7 and 14 regional samples in M9.

See Supplementary File 3

Supplementary Table 3G: Overlap between (1) genes mutated in M7, M9, or either, shown in rows, and (2) six lists of cancer-related genes, shown in columns. Shown are the number of overlapped genes and its percentage of the corresponding cancer-related gene list. Note the higher percent of overlap with the ESCC- and SCLC-genes.

See Supplementary File 3

Supplementary Table 4A: Mutations selected for DTS for M7. Shown are 68 mutations targeted for resequencing. Columns A-E are chromosome, position, nucleotide change, Refseq gene name, and annotated function change. F to K are the genotypes in individual samples.

See Supplementary File 4

Supplementary Table 4B: Mutations selected for DTS for M9. Shown are 131 mutations targeted for resequencing. Columns A-E are chromosome, position, nucleotide change, Refseq gene name, and annotated function change. F to M are the genotypes in individual samples.

See Supplementary File 4

Supplementary Table 4C: WES-DTS comparison for 408 observed mutations in M7 samples. Fisher's exact test was used to test if two binomial proportions from WES and DTS are significantly different or not in M7. Columns A-O are chromosome, position, sample ID, nucleotide change in WES, Refseq gene names, on-off status in WES, nucleotide change in DTS, number of mutation allele reads in WES, number of total reads in WES, number of mutation allele reads in DTS, number of total reads in DTS, allele frequency in WES, allele frequency in DTS, Fisher's exact test p value, and the Odds Ratio.

See Supplementary File 4

Supplementary Table 4D: WES-DTS comparison for 1,048 observed mutations in M9 samples. Content is similar to S4C, and the format is the same.

See Supplementary File 4

Supplementary Table 4E: Summary of validation results in M7. Shown are counts and percentages of validated mutations in six samples from M7.

See Supplementary File 4

Supplementary Table 4F: Summary of validation results in M9. Shown are counts and percentages of validated mutations in eight samples from M9.

See Supplementary File 4

Supplementary Table 5A: Inferred copy number of alterations in M7. Shown are 1,544 copy number segments called by *ASCAT* in M7 samples. Columns are regional sample name, chromosome, start position of the segment, end position, copy number of the major allele, and copy number of the minor allele.

See Supplementary File 5

Supplementary Table 5B: Inferred copy number of alterations in M9. Shown are 3,812 copy number segments called by *ASCAT* in M9 samples, in the same format as in S5A.

See Supplementary File 5

Supplementary Table 5C: Comparison of total length of CNAs for different CNA types across 11 M7 sample. CNA types in column A are defined by nMajor and nMinor combinations. For example, “1-1” in row-5 is the normal diploid state. From columns B and C, every two columns show a sample's total length and fractional genome coverage for each CNA type.

See Supplementary File 5

Supplementary Table 5D: Comparison of total length of CNAs for different CNA types across 14 M9 sample, in the same format as Supplementary Table 5C.

See Supplementary File 5
